# Home-like care: Collaboration between parents and nurses in everyday situations when children are hospitalized

**DOI:** 10.1177/13674935221149778

**Published:** 2023-01-06

**Authors:** Hildegunn Sundal

**Affiliations:** Faculty of Health Sciences and Social Care, 5562 Molde University College, Molde, Norway

**Keywords:** Child, hospitalized, collaboration, responsibility, care, everyday situation, nurses, parents

## Abstract

When children are hospitalized, parents and nurses need to collaborate. This study aims to investigate how parents and nurses experience collaborating and sharing responsibilities and tasks when providing home-like care for hospitalized children in everyday situations. This qualitative study used a hermeneutic phenomenological approach with observations and interviews and was conducted in a general medical pediatric unit. Twelve parents of eleven hospitalized children between the ages of 1 and 6 years with various medical diagnoses and seventeen nurses who cared for those children participated in the study. Parents and nurses collaborated and shared responsibilities and tasks to provide home-like care for hospitalized children in everyday situations by making mealtimes seem familiar, maintaining customary sleeping patterns, adapting washing and dressing routines, and facilitating play and activity. Parents and nurses collaborated to maintain a familiar rhythm in an unfamiliar environment to enhance the children’s well-being. The nurses’ degree of involvement in the children’s everyday situations varied from little to moderate to strong, with parents assuming the main responsibilities. Nurses’ involvement in children’s everyday situations was variable, depending on the complexity of the situations.

## Introduction

When children are hospitalized, parents play a crucial role in their care (Sundal and Vatne, 2020). In these situations, collaboration between parents and nurses to provide home-like care in everyday situations is of great importance (Sundal and Vatne, 2020). Specifically, home-like care ensures that, despite an unfamiliar environment, the child continues to experience each day in a way that resembles the everyday life as closely as possible (Sundal and Vatne, 2020). In addition, family-centered care offers a way for collaboration between parents and nurses (Aarthun et al., 2018; Kurnia et al., 2022; Uniacke et al., 2018) and the involvement of parents in child’s care through a partnership with nurses (Christian, 2016; Clerke et al., 2017), to ensure a respectful relationship between nurses and parents (Ridgway et al., 2021). Indeed, partnership with parents or other family members improves the quality of care for children (Christian, 2016), and family engagement contributes to a mutually beneficial partnership between families and health care team members (Barratt et al., 2021; Jerofke-Owen et al., 2022; Rafferty and Sullivan, 2017). Engagement strategies range from more passive strategies, such as allowing and encouraging families to be present at the bedside, to more active strategies aimed at promoting mutual and reciprocal nurse–patient interactions (Jerofke-Owen et al., 2022). Parental support and empowerment have been emphasized as prerequisites for their involvement and engagement in the daily care of their child (Ashcraft et al., 2019).

Previous studies have described how parents perform daily or basic care for their hospitalized child (Abdelkader et al., 2016; Coyne, 2015; Harder et al., 2016), and daily care tasks were delegated to families, regardless of their child’s clinical condition (de Macedo et al., 2017). Indeed, parents may recognize that basic care is their responsibility, but they need assistance to manage this new situation (Curtis and Northcott, 2017). The responsibility of parents caring for their child in hospital needs to be supported by nurses, but nurses also need to emphasize their limitations in providing basic care for children due to time constraints (Coyne, 2015). Parents are expected to collaborate with professionals (Harder et al., 2016), and they need support from nurses (Curtis and Northcott, 2017). Furthermore, parents have reported a desire to be included in decision-making processes regarding the care of their child (Walker-Vischer et al., 2015), and that it is important to them to avoid situations where they feel that they are making undue demands on nurses’ time (Curtis and Northcott, 2017). In hospital, parents’ basic care tasks tend to include bathing, dressing, feeding, mobilizing, and comforting their child (Coyne, 2015). Moreover, care activities that parents want to participate in with their hospitalized child include mealtimes, toileting and hygiene care, massage and skin care, play activities, and regulating their temperature and sleep (Çamur and Sarıkaya Karabudak, 2021).

## Aim

To investigate how parents and nurses experience collaborating and sharing responsibilities and tasks when providing home-like care for hospitalized children in everyday situations.

## Method

This qualitative study used a hermeneutic phenomenological approach (Van Manen, 1990, 2014) to investigate the lived experiences of parents and nurses and gain an understanding of their collaboration through interviews. According to Van Manen (1990, 2014), a phenomenological description is an interpretation of descriptions and meanings, and interpreting such descriptions entails some elements of a hermeneutic approach. This hermeneutic interpretation is based on understanding the whole in terms of the parts and the parts in terms of the whole. Specifically, to gain an understanding of parents and nurses’ perspectives on collaboration, both were observed while collaborating and then interviewed (Brinkmann and Kvale, 2015; Hammersley and Atkinson, 2007, 2019; Wadel, 1991). The empirical findings were considered in relation to the theory of caring, with a focus on dwelling, a human’s way of existing in this world (Martinsen, 1989, 2001).

### Participation

This study was conducted in a general medical pediatric unit in a Norwegian hospital, which has a capacity for 12 children and is a child-friendly environment. Inclusion criteria for participation in this study were as follows: the parents had to be present throughout the children’s hospital stay, children were at the start of their admission and would most likely remain hospitalized for two or more days, children were between 1 and 6 years old, parents spoke Norwegian as their first language to simplify communication and prevent misunderstandings, and children were neither critically nor terminally ill; this avoided any challenging situations related to serious illness and probable death.

### Ethical considerations

This study was approved by a regional research ethics committee ( 4.2006.3865/4.2007.1097) and the Ministry of Health and Care Services ( 07/3088-14.06.2007). This study was also reported to a privacy protection committee (04.06.2007-16697/JE). Written consent was obtained from the participating parents and nurses by the medical pediatric unit’s head nurse, and they were informed of their rights (including confidentiality and voluntariness). Parents provided consent on behalf of their children. Ethical considerations included recognition of the vulnerability of both parents and children in a situation where children may suffer in an unfamiliar environment. The child’s lack of maturity also makes them more vulnerable (Solbakk, 2015). After the researcher introduced herself to the parents and played with the child, if possible, to become acquainted with them, the author then assumed a background position to not disturb any interactions between nurse, parents, and the child and left, together with the nurses once the interactions ended.

### Data collection

The author acted as a partial participant observer and sometimes participated in care, such as passing equipment to a nurse (Hammersley and Atkinson, 2019; Wadel, 1991). Each week, one group, consisting of a child, their parent(s), and a nurse, was observed, which involved shadowing every morning shift nurse responsible for the child and during some afternoons, until discharge (two to four dayshifts per child). The author remained nearby at the start of each observation period, including participating in small talk, and later moved into the background. Descriptive and reflective notes were written shortly after observing, with descriptions focusing on personal relationships and movements, conversation and play, and the performance of practical tasks related to sleep, mealtimes, washing, and dressing. Qualitative research interviews (Brinkmann and Kvale, 2015) were conducted with the parents and nurses upon discharge, with a focus on participants’ experiences of collaboration during activities such as sleep, mealtimes, washing, dressing, and playing, based on the observed situations. Most of the interviews were conducted in the hospital before the children and parents left; however, one parent was interviewed at home a day after discharge and another by telephone 7 days after discharge. All interviews (45–90 min) were conducted after observations had been done and were audio-taped and transcribed verbatim.

### Data analysis

A thematic analysis, inspired by Max Van Manen’s holistic, selective, and detailed approaches, was performed (Van Manen, 1990, 2014). First, interview transcripts and notes from each child were holistically read to capture overall meaning. Second, texts were selectively read to determine preliminary themes related to the phenomenon under investigation; this selective reading was done to discover aspects or qualities that define the phenomenon. Third, texts were read in detail, and sentences and phrases were clustered into preliminary themes and subthemes.

### Findings

In total, twelve parents (nine mothers and three fathers) of eleven hospitalized children and seventeen female registered nurses participated in this study. The participating nurses were caring for 11 children when observations were performed. Data were gathered over 4 months through a combination of observation (11 children, 12 parents, and 17 nurses) and qualitative research interviews. In addition, thirteen nurses and all parents were interviewed; four nurses were not interviewed due to practical considerations. Five children had been admitted with acute conditions, and six were planned admissions. They had various medical diagnoses, and four had congenital chronic medical disorders. Two children were 1 year old, four were 2 years old, four were 3 years old, and one was 6 years old, and there were three boys and eight girls. Hospitalization lasted between 2 to 4 days, and one child was readmitted for 1 day. One main theme and four subthemes emerged through comparing preliminary themes and subthemes from the twelve parents, eleven hospitalized children, and seventeen nurses’ participation.

### Home-like care in everyday situations

The theme, home-like care in everyday situations for hospitalized children, was characterized by nurses and parents collaborating in performing tasks and maintaining a familiar rhythm in an unfamiliar environment. These actions were delineated into four subthemes: making mealtimes seem familiar to children, maintaining customary sleeping patterns, adapting washing and dressing, and facilitating play and activity. The situations involved varying degrees of collaboration between parents and nurses, and the nurses alternated between more active or passive roles when providing for everyday needs.

Hospitalization in an unfamiliar environment with a different rhythm poses a challenge to children’s familiar rhythms. Parents and nurses achieved the goal of maintaining a familiar rhythm by minimizing and limiting unfamiliar variables in the children’s everyday situation. Specifically, nurses adapted and redefined framework conditions to safeguard activities, tasks, and routines that are familiar to both children and parents. In addition, parents performed caring activities and tasks to promote a familiar rhythm and compensate for an altered rhythm. Parents remained with their children continuously, and this action in itself contributed to a feeling of familiarity. Parents also initiated and performed well-known activities and tasks that were facilitated by nurses with the intention of individualizing everyday situations to promote a familiar rhythm in relation to meals, sleep, washing and dressing, and play and activities. Parents were the initiators and were mainly responsible for asking for help. However, parents sometimes found it challenging to ask for help because the nurses were not available or were too busy.

Nurses and parents collaborated to maintain a familiar rhythm. However, the nurses varied their degree of involvement from little to moderate to high to meet the everyday needs, and different variables contributed to the degree of nurse involvement. For example, the severity of disease, children’s age, parents’ experience with hospitals, and how the children experienced hospitalization resulted in varying degrees of nurse involvement. Specifically, children who were more severely ill and younger children were associated with greater nurse involvement. Sometimes nurses were nearby, but, at other times, they were not. Moreover, their participation varied between helping, supporting, and taking over or retreating or maintaining their distance, if necessary. Their involvement changed constantly as they attempted to maintain a familiar rhythm in the everyday situations and did what was needed to safeguard the children’s well-being in terms of security and predictability.

### Making mealtimes seem familiar

Making mealtimes feel familiar in an unfamiliar environment contributed to predictability for children. Parents performing the activities related to mealtimes as they do at home was important in the creation of a home-like space in hospital, which involves unknown rooms and different mealtimes. Nurses responded to this difference by adapting the hospital framework conditions to suit families’ wishes; for example, they considered whether the family wanted to eat in their own room or a dining room and whether or not they wanted to collect the food themselves. A nurse explained this adaptation as follows:

*“I tell them when it is mealtime, when food is prepared, and I offer them food. They can choose whether to eat in the dining room or their own room.” (Isabella’s nurse)*

With this new framework in place, for example, to eat in their own room or a dining room, parents could perform familiar activities and create a home-like space, as noted in the following statement from one participating mother:

*“I went to get food myself, except when I could not find out when the meal was served, and we ate in the room.” (Isabella’s mother)*

Nurses ensured that parents were informed of mealtimes and adapted the framework of the mealtimes to enable the parents to be self-reliant.

The children’s diseases and treatment can affect mealtimes and present new challenges, such as children requiring help with eating, and, thus, familiar activities and tasks sometimes had to be adapted by parents with such considerations in mind. Nurses also adapted or redefined framework as necessary; for example, if a child needed a different type of food due to a lack of appetite. In some situations, nurses were more directly involved by interacting with the children to clarify their needs or remaining during mealtime to clarify the framework, and then retreated to allow for parents to carry out familiar activities. In one example situation, mealtime was redefined by a child’s request to not eat in bed. Specifically, a child who was hospitalized regularly was in bed receiving an infusion when lunch was served and wanted to eat at a table; this wish was taken into account by the nurse and the child’s father. According to the nurse

*“This child with frequent admissions needs to be listened to. A lot is at stake.” (Olivia’s nurse)*

Furthermore, their father emphasized that:

“*Children require regular routines at meals in hospital.” (Olivia’s father)*

The degree of nurses’ involvement in mealtimes varied, and they worked to facilitate and adapt the framework before finally redefining it. They adjusted how they collaborated, to which extent they were involved, and how they reacted to each individual situation, as exemplified by accommodating the aforementioned child who did not want to eat in bed.

Nurses were less involved when parents were self-reliant and more moderately involved when parents were inexperienced with hospitalization or if the children required more facilitation due to their age. Greater levels of involvement occurred when the children’s disease or treatment affected mealtimes. In such cases, the need to adapt situations to individual patients’ needs increased, and disrupting routine was necessary to accommodate issues such as a child’s desire for specific or age-appropriate food.

### Maintaining customary sleep patterns

When parents carry out activities and tasks in a similar rhythm to what is maintained at home, children feel a sense of safety and predictability. Sleeping involved a private space for children and parents, so parents arranged furniture according to familiar rituals that would normally be carried out at home. In addition, parents and nurses collaborated to compensate for a lack of framework conditions, such as the bed and room that the child is familiar with at home. Nurses respected boundaries by creating a home-like space for parents to carry out familiar activities and by allowing each family their own space. One of the participating nurses clarified it in this statement:

“*We should not interfere when a mother has her routines. We will not take over when mother does what she does best. One must retreat.” (Leo’s nurse)*

Nurses maintained their distance to allow parents to carry out their routines and activities when framework conditions were in place. For example, they reported maintaining a distance after a parent borrowed a pram when their room was not available on arrival and the child needed to sleep. Parents used rhythm and predictability to create a home-like space, and, therefore, unfamiliar elements were reduced or removed.

Some children’s customary sleeping rhythm had been disturbed, and they experienced an increased need for sleep, which had to be accommodated as an extended need. A frequent parenting activity in home-like spaces involves allowing children to sleep with their parents. In such situations, nurses involved themselves by, for example, entering a home-like space with caution and then choosing to retreat and maintain their distance. Nurses identified needs and boundaries within home-like spaces and closed-off areas at intervals to limit external disturbances. Indeed, one of the participating mothers said that

*“My responsibility is to take care of the child, try to normalize things, and see that the child follows routines like at home.” (Amelia’s mother)*

### Adapting washing and dressing

Both parents and nurses regarded washing and dressing of children in an unfamiliar hospital environment as the parents’ responsibility. Since there is no familiar bathroom, nurses had to adapt equipment so that parents could wash and dress their children appropriately, thus enabling parents to carry out familiar activities and contribute to creating a home-like space. A familiar washing and dressing rhythm was created by parents, based on their children’s expectations. In this way, a home-like space was defined by parents’ familiar activities and their closeness to their children.

The parents’ needs for help varied, and some parents had greater difficulty than others in asking for help. Nurses were present during certain periods with varying degrees of involvement and were responsible for facilitation. They also maintained a background presence for varying periods of time and helped by retrieving required equipment, for example, a need for baby equipment, and to improve framework conditions. Nurses supported parents; their involvement and collaboration were minimal in some cases and moderate in others, as demonstrated by this following quote from a mother in a nurse’s presence:

“*I have the main responsibility and nurse helps me… I am the mother, the caregiver.” (Leo’s mother)*

In addition, a nurse commented as follows:

“*I am just trying to be there for the mother and help her with what she needs. Mother knows she needs help.” (Leo’s nurse)*

In some more demanding situations, washing and dressing of children were affected by their illness and treatment and required finding a solution through collaboration. Even when nurses attempted to adapt a framework to some extent, certain complex situations required the nurses to perform some activities involving children directly. In addition, in some situations, nurses alternated between being actively involved and remaining a background presence. When necessary, parents and nurses swapped roles, but this affected the home-like quality of spaces. Familiar rhythms then broke down, which could make the children feel less safe. This situation is exemplified by a case involving diaper change of a child with diarrhea as result of a nasogastric treatment administered for constipation. Although the mother asked for help every time and the nurses obliged, the child wanted her mother to do it, and the nurse also asked mother to do it every time. In this case, a child’s wish was temporarily ignored, and, according to a nurse,

“*The child felt more comfortable when mother did it and asked the mother to do it, and I asked mother to do it.” (Sara’s nurse)*

Conversely, the mother gave the following statement:

*“I have never been more tired. It was very good that the nurse did it.” (Sara’s mother)*

### Facilitating play and activity

The parents’ main responsibility toward children affected by symptoms and treatment was to create a home-like space in which children could play and engage in their own activities between meals, sleeping, and participating in other hospital activities. At times, this was a daunting task for the parents. For instance, children wanting to play or be active during infusions is a common challenge. Moreover, parents often experienced the challenge of their children having to fast prior to a medical procedure performed under anesthesia, as in the following example. A father experienced this issue:

“*I could not get out of bed. It was the only place I could calm the child. The situation was stressful. We waited a long time, and the child had to go without food.” (Adam’s father)*

A nurse commented on a fasting situation as follows:

“*It was a long day, especially with respect to the fasting… It was very wise to pick up a movie and try to come up with things to play with.” (Adam’s nurse)*

The nurses’ support, help, and facilitation were also required; for example, they helped pick up toys and improved framework conditions for children’s play.

## Discussion

This study aimed to identify how parents and nurses experience collaborating and sharing responsibility and tasks when providing home-like care. Accordingly, findings in this study demonstrate that this collaboration is characterized by involving both nurses and parents. To maintain parents’ familiar rhythm, activities, and tasks, both groups alternated between being active or passive during this collaborative relationship, and the degree of nursing involvement in everyday situations ranged from little to moderate to strong, based on the extent of the children’s illness and treatment. Overall, this involvement and collaboration contributed to creating a home-like space for children in everyday situations and produced a feeling of continuity with home. Making mealtimes familiar to children, maintaining customary sleeping patterns, adapting washing and dressing, and facilitating play and activities are everyday situations that required nurses and parents to collaborate during children’s hospital stays.

Norwegian nurse and philosopher Martinsen (1989) discussed dwelling in her theory of care following Heideggerian tradition. A human’s way of existing in this world involves dwelling; dwelling is feeling at home and inhabiting this world with a feeling of security and belonging when the world is recognizable and predictable. Furthermore, creating habits is a part of dwelling, and we shape our activities into habits with a rhythm. A body’s habits in a house gather and order their world through their rhythms. Even when an individual moves from one house to another, an inhabitable and recognizable world emerges through physical space, including an order and rhythm of place, its objects and interiors, and activities that involve interacting with those objects and interiors (Martinsen, 1989, 2001).

A hospital as a public house and place is guided by a general, universal order and rhythm rather than an individualized order and rhythm to ensure patient flow. When an ill individual inhabits a hospital with its general order and rhythm, illness and treatment demand an individually adapted structure in each patient’s room, which nurses safeguard through professional responsibility (Martinsen, 2001).

The nurses’ professional responsibility is to individualize the children’s everyday situations to preserve parents’ familiar activities, work tasks, and routines. However, parents’ routines often differ from a hospital’s general order and rhythm. Children’s illnesses and treatments influence everyday situations and require a stronger degree of individualization. In this study, some tension was found in the collaborations between parents and nurses, the hospital’s general order and rhythm, and the safeguarding of individualization (Martinsen, 2001). Concurrently, according to Martinsen (2001), a general order and rhythm of hospitals are preconditions that individualize children’s everyday situations and safeguard their individual needs.

### Collaboration on individualization of general order

Nurses individualize a hospital’s general order by facilitating, adapting, and redefining framework conditions. For example, the parents of the youngest children were offered extra equipment to adapt the care to suit the specific needs of the children in new situations and adapt framework conditions to the families’ wishes to make mealtimes feel familiar. According to Martinsen, individualizing and adapting framework conditions in children’s everyday situations facilitate the creation of a feeling of home in hospital (Martinsen, 2001). This study’s findings align with those of previous studies into parents’ main responsibilities in providing home-like care, also described as daily or basic care (Abdelkader et al., 2016; Coyne, 2015; de Macedo et al., 2017; Harder et al., 2016). Basic care consists of bathing, dressing, feeding, mobilizing, and comforting children (Coyne, 2015), and Çamur and Sarıkaya (2021) had similar findings regarding these actions that constitute basic care and that they are mainly the responsibility of the parents. These situations relate to mealtimes and washing, dressing, and sleeping situations involved in home-like care. This study contributes to knowledge regarding how nurses collaborate with parents and individualize care for each patient by facilitating, adapting, and redefining framework conditions to address each child’s everyday situations and needs. These findings partially coincide with those of a previous study regarding the support that parents receive during their involvement and engagement in the daily care of their hospitalized children (Ashcraft et al., 2019) and are also in accordance with previous work reporting on collaborative partnerships that parents and nurses engage in to promote quality in children’s care (Christian, 2016).

### Collaboration on individualizing general rhythms

Nurses and parents individualize and disrupt a hospital’s general rhythm by adapting those rhythms and routines to those of the children, relating to mealtimes, care, sleep, and play and activity situations. Parents depend on nurses’ abilities to identify their children’s individual needs; for example, a nurse may allow a child to eat at a table if they want to, and, if this is appropriate, nurses facilitate children playing and engaging in their own activities when a child has to fast prior to a medical procedure performed under anesthesia (Martinsen, 2001). The importance of play to enhance children’s well-being is consistent with previous studies (Claus et al., 2021; Godino-Iáñez et al., 2020; Li et al., 2016; Ullan and Belver, 2019), and children’s play strategies are understood as a way to entertain and distract hospitalized children (De Paula et al., 2019).

According to Martinsen (2001), the general rhythm of hospitals challenges how parents and nurses collaborate to safeguard a child’s individualized rhythm in everyday situations to enhance their sense of security. For example, a hospital’s general rhythm, routines, and activities may cause a nurse to prefer that a child eats in bed despite their wish to eat at a table and to not maintain a distance when a child’s customary sleeping rhythm is disturbed. A hospital’s general rhythm is influential through routines and activities embodied by nurses. However, nurses may use their power positively to adapt and individualize routines to allow for parents’ familiar activities and children’s rhythms.

According to Martinsen (2001), nurses’ routines may have to submit to a hospital’s rhythm. In those cases, nurses may use their power negatively; for example, a nurse may not accommodate a child’s wish to eat at a table. This study’s findings on nurses’ involvement in children’s everyday situations where nurses individualize their care by facilitating and collaborating with parents partially align with previous findings regarding the importance of empowering parents to participate in their child’s care (Ashcraft et al., 2019) and in their desire to participate in decision-making (Walker-Vischer et al., 2015). These findings are also in line with previous work that has demonstrated that parents are expected to collaborate with professionals (Harder et al., 2016), but correlate less with previous results that have shown that parents may perceive nurses as inaccessible (Coyne, 2015). Finally, active strategies aimed at promoting nurse–patient interactions (Jerofke-Owen et al., 2022) correspond to dynamic collaboration between nurses and parents in everyday situations, as shown by findings in this study.

### Collaboration on home-like spaces

Facilitating and adapting structures help to limit and define what Martinsen (2001) refers to as a fictive space in a room, which is a home-like space created at mealtimes, sleep time, care time, and play and activity time, that makes children feel safe. Indeed, creating a smaller world through developing a fictive space in a room may simplify the process of caring for a sick child.

According to Martinsen (2001), a fictive room is defined by activities, habits, and rhythms for interacting with objects and interiors as framework conditions and induces a feeling of safety. Home-like spaces for the everyday situations of children protect sick children, and these spaces are created when parents carry out familiar activities and tasks and nurses individualize everyday situations. In addition, parents can become more self-reliant in everyday situations when a home-like space is defined and limited.

Parents have an embodied rhythm that corresponds to their children’s rhythm, and they all inhabit a home-like space with a sense of closeness when performing familiar activities. Children’s illnesses and treatment often entail further involvement by nurses, such as adapting framework conditions to facilitate eating at a table and different types of food to tempt a child who has a poor appetite. Over time, a child affected by illness and treatment who encounters nurses who take responsibility for certain activities may experience a disruption in the familiar rhythm normally embodied by parents. For example, a nurse may take over and change a child’s diaper. Furthermore, parents and nurses may change roles, with parents either relinquishing control or having it taken from them in certain situations, which can affect a home-like space with its familiar rhythm. Furthermore, this issue may be reinforced by disagreements on divisions of chores or the nurses’ degree of involvement in care. Ultimately, a child may feel unsafe because of a familiar rhythm fading or disappearing (Martinsen, 2001).

In home-like care, nurses may be either closely involved with patients or involved at a distance, and their degree of involvement varies greatly. An example of this is a nurse maintaining a distance after a parent borrowed a pram for a child to sleep in when their room was not available on arrival. The dynamic collaboration identified in this study may be characterized by a constantly changing balance in how the care is shared. This finding is comparable to a finding of Harder et al. (2016), which has shown that healthcare professionals understand that care situations with children is an ambiguous challenge, since the characteristics of each situation depend on the participants involved, namely, the children, parents, and professionals.

### Strengths and limitations of this study

This study was conducted in one small general medical pediatric unit of a Norwegian hospital in one geographical area with children with different medical diagnoses. Therefore, while the findings are of value in similar contexts and cultures, a possible limitation of this study is that it was conducted in a single center. The strengths of the study are the use of partial participant observation and qualitative interviews, which provided several avenues to generate insights into the daily lives of parents and children collaborating with nurses in a hospital. However, the children were not invited to participate, so the study does not include the direct perspectives of children. Interactions between nurses, parents, and children may have been affected by the researcher’s presence.

### Implications for practice

This study can influence nursing practice by alerting professionals of the need to individualize each child’s everyday situation in hospital, especially in situations characterized by illness and the required treatment of the child. Nurses’ adaption of framework conditions to meet the parents’ needs is of great importance in the care of sick children in everyday situations.

## Conclusion

This study of home-like care demonstrates how parents and nurses collaborate and which tasks they collaborate on to maintain a familiar rhythm in children’s everyday situations in hospital. The degree to which nurses collaborated with parents varied from little to moderate to strong, based on the complexity of the everyday situations. Nurses facilitated and individualized framework conditions in everyday situations and alternated between being closely involved with the children and maintaining some distance when performing their tasks to maintain a familiar rhythm and assist parents to create a home-like space in those everyday situations to enhance their children’s well-being. Parents represent continuity for children and are primarily responsible for the home-like care to allow their children to feel safe.
